# Supplementary material for: Diet induces parallel changes to the gut microbiota and problem solving performance in a wild bird
Source: Sci Rep. 2020 Nov 27;10:20783. doi: 10.1038/s41598-020-77256-y (PMC7699645; doi:10.1038/s41598-020-77256-y)
Supplement: Supplementary file 2 — Supplementary Information 2. [file 41598_2020_77256_MOESM2_ESM.docx]

Supplementary Materials

Title: “Diet induces parallel changes to the gut microbiota and problem solving performance in a wild bird”. Authors: Gabrielle Davidson^a,b,*^ , Niamh Wiley^c,d^, Amy C. Cooke^a^, Crystal N. Johnson^c,d^, Fiona Fouhy^c,d^, Michael S. Reichert^a, e^, Iván de la Hera^a^, Jodie M.S. Crane^a,f^, Ipek G. Kulahci^a^, R. Paul Ross^c,d^, Catherine Stanton^c,d^, John L. Quinn^a,*^

* Correspondence: Gabrielle Davidson email: gd339@cam.ac.uk; John Quinn email: j.quinn@ucc.ie.

Tel: +44(0)1223 747321

1. School of Biological, Earth and Environmental Sciences, Distillery Fields, North Mall, University College Cork, Cork, Ireland.
2. Department of Psychology, Downing Street, University of Cambridge, Cambridge, UK.
3. APC Microbiome Ireland, University College Cork, Cork, Ireland.
4. Teagasc Food Research Centre, Moorepark, Fermoy, Ireland.
5. Department of Integrative Biology, Oklahoma State University, USA
6. Kākāpō Recovery Programme, Department of Conservation, 7th Floor, 33 Don Street, Invercargill 9810, New Zealand.


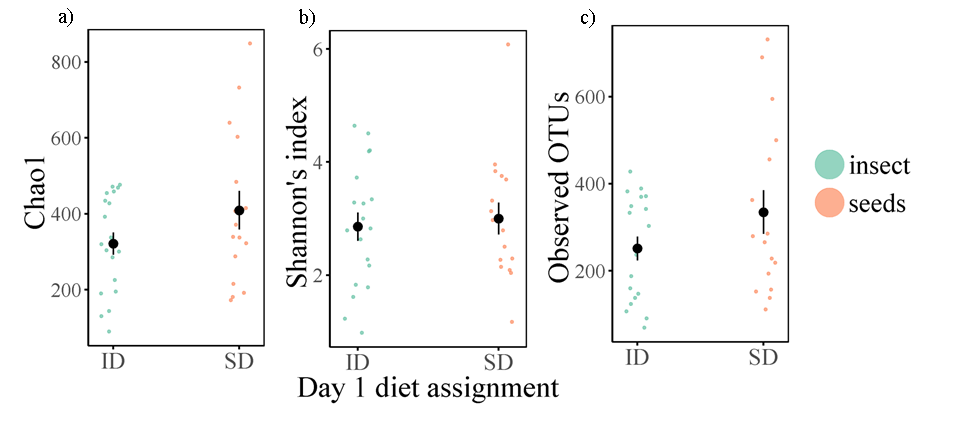


Figure S1. Alpha diversity for samples taken on day 1 for each dietary assignment. Linear models with microbiome as the response variable and diet as a fixed effect showed the gut microbiome did not differ between birds on Day 1 according to their dietary assignments: (a) Chao 1: Estimate (Est.)=87.80, slope (β)= 56.62, t=1.55, p=0.131; (b) Shannon’s index: Est.=0.14, β= 0.38, t=0.382, p=0.705; (c) observed OTUs: Est.=83.65, β= 54.65, t=1.53, p=0.135.

Beta diversity did not differ at day 1 between the two dietary assignments (Jaccard: R2=0.03, p=0.61; weighted unifrac: R2=0.04, p=0.22; unweighted unifrac: R2=0.04, p=0.23. Nor did phylum-level abundance: Proteobacteria: Est.=-0.001, β=0.11, t=0.012, p=0.99; Firmicutes: Est.=-0.02, β=0.05, t=-0.464, p=0.65; Bacteroidetes: Est.=0.00, β=0.002, t=-0.289, p=0.77; Actinobacteria: Est.=0.001; β=0.03, t=0.05, p=0.96; Tenericutes: Est.=-0.07, β=0.07, t=-0.97, p=0.34.

Table S1. Estimated nutritional content of food types provided in dietary treatments. Percentages of protein, fat and fibre content attained from online resources.

| Food | Protein | Fat | Fibre |
| --- | --- | --- | --- |
| mealworm larvae | 18.70% | 13.40% | 2.50% |
| waxworm larvae | 14.10% | 24.90% | 3.40% |
| peanuts | 25.80% | 49.20% | 8.50% |
| sunflower hearts | 18.33% | 46.67% | 10% |
| beef suet | <1% | >95% | 0 |

Table S2. GLMM outputs from models testing dietary effects on problem solving performance and gut microbiome. PSP = Problem Solving Performance.

| **Model** | **subjects** | **total observations** | **Fixed effects** | **β±SE** | **z/t** | **p** |
| --- | --- | --- | --- | --- | --- | --- |
| **Diet on PSP** | 36 | 71 | intercept | -0.88±0.52 | -1.70 | 0.09 |
| Minimal model | 36 | 71 | seed diet | 1.43±0.66 | **2.20** | **0.03** |
|  | 36 | 71 | age (adult) | -1.32±0.68 | -1.93 | 0.053 |
|  | 36 | 71 | experiment day | -1.23±0.64 | -1.93 | 0.054 |
| dropped terms | 36 | 71 | sex (male) | -0.20±0.68 | -0.30 | 0.76 |
|  | 36 | 71 | habitat | -0.06±0.59 | -0.10 | 0.92 |
|  | 36 | 71 | diet*habitat | 1.09±1.22 | 0.89 | 0.37 |
|  | 36 | 71 | diet*experiment day | 17.6±1445 | 0.01 | 0.99 |
| **Diet on Chao1** | 36 | 61 | intercept | 18.5±0.70 | 26.54 | 0.00 |
| Minimal model | 36 | 61 | insect diet | -3.44±1.37 | **-2.51** | **0.02** |
|  | 36 | 61 | seed diet | 0.09±1.30 | 0.07 | 0.94 |
| dropped terms | 36 | 61 | habitat (urban) | 0.67±1.90 | 0.61 | 0.55 |
|  | 36 | 61 | sex (male) | -0.45±1.08 | -0.41 | 0.68 |
|  | 36 | 61 | age (adult) | -0.34±1.10 | -0.31 | 0.75 |
|  | 36 | 61 | diet*habitat | 3.07±2.11 | 1.45 | 0.16 |
| **Diet on Shannon's index** | 36 | 61 | intercept | 2.93±0.20 | 14.75 | 0.00 |
| Minimal model | 36 | 61 | insect diet | -0.75±0.37 | -2.02 | 0.06 |
|  | 36 | 61 | seed diet | 0.01±0.35 | 0.01 | 0.99 |
| dropped terms | 36 | 61 | habitat (urban) | -0.14±0.33 | -0.42 | 0.68 |
|  | 36 | 61 | sex (male) | 0.22±0.32 | 0.67 | 0.51 |
|  | 36 | 61 | age (adult) | 0.01±0.33 | 0.02 | 0.98 |
|  | 36 | 61 | diet*habitat | 0.92±0.64 | 1.44 | 0.16 |
| **Diet on observed species** | 36 | 61 | intercept | 5.5±0.01 | 58.80 | 0.00 |
| Minimal model | 36 | 61 | insect diet | -0.32±0.19 | -1.74 | 0.095 |
|  | 36 | 61 | seed diet | 0.08±0.18 | 0.43 | 0.67 |
| dropped terms | 36 | 61 | habitat (urban) | 0.17±0.14 | 1.19 | 0.24 |
|  | 36 | 61 | sex (male) | -0.01±0.14 | -0.10 | 0.92 |
|  | 36 | 61 | age (adult) | -0.07±0.15 | -0.49 | 0.62 |
|  | 36 | 61 | diet*habitat | 0.42±0.28 | 1.52 | 0.14 |
| **Diet on Proteobacteria** | 36 | 61 | intercept | -0.42±0.43 | -0.97 | 0.33 |
| Minimal model | 36 | 61 | insect diet | 1.8±0.87 | **2.02** | **0.04** |
|  | 36 | 61 | seed diet | -0.19±0.66 | -0.28 | 0.78 |
|  | 36 | 61 | age (mature) | 1.05±0.57 | 1.84 | 0.07 |
| dropped terms | 36 | 61 | sex (male) | -0.02±0.6 | -0.03 | 0.98 |
|  | 36 | 61 | habitat (urban) | 0.35±0.57 | 0.61 | 0.54 |
|  | 36 | 61 | diet*habitat | -0.29±1.11 | -0.26 | 0.80 |
| **Diet on Bacteroidetes** |  |  | intercept | -6.23±0.30 | -20.60 | 0.00 |
| Minimal model | 36 | 61 | insect diet | 1.32±0.59 | **2.25** | **0.03** |
|  | 36 | 61 | seed diet | 0.56±0.56 | 1.01 | 0.32 |
|  | 36 | 61 | habitat (urban) | 0.82±0.46 | 1.80 | 0.08 |
| dropped terms | 36 | 61 | sex (male) | 0.58±0.45 | 1.29 | 0.20 |
|  | 36 | 61 | age (adult) | 0.09±0.47 | 0.20 | 0.84 |
|  | 36 | 61 | diet*habitat | 1.54±0.92 | 1.68 | 1.00 |
| **Diet on Firmicutes** |  |  | intercept | -3.00±0.23 | -12.93 | 0.00 |
| minimal model | 36 | 61 | age (adult) | -0.63±0.34 | -1.85 | 0.07 |
|  | 36 | 61 | insect diet | -0.39±0.42 | -0.92 | 0.37 |
| dropped terms | 36 | 61 | seed diet | 0.15±0.40 | 0.39 | 0.70 |
|  | 36 | 61 | habitat (urban) | 0.07±0.35 | 0.20 | 0.84 |
|  | 36 | 61 | sex (male) | 0.02±0.37 | 0.66 | 0.52 |
|  | 36 | 61 | diet*habitat | 1.16±0.69 | 1.69 | 0.10 |
| **Diet on Tenericutes** |  |  | intercept | -5.34±0.45 | -11.97 | 0.00 |
|  | 36 | 61 | insect diet | 0.24±0.80 | 0.30 | 0.15 |
|  | 36 | 61 | seed diet | 1.11±0.76 | 1.46 | 0.15 |
|  | 36 | 61 | habitat (urban) | 0.01±0.74 | 0.02 | 0.99 |
|  | 36 | 61 | sex (male) | -1.16±0.71 | -1.63 | 0.12 |
|  | 36 | 61 | age (adult) | -0.15±0.74 | -0.20 | 0.84 |
|  | 36 | 61 | diet*habitat | -0.84±1.53 | -0.55 | 0.59 |
| **Diet on Actinobacteria** |  |  | intercept | 0.00±0.00 | 1.18 | 0.24 |
|  | 36 | 61 | insect diet | 0.00±0.00 | 1.48 | 0.15 |
|  | 36 | 61 | seed diet | 0.00±0.00 | -0.53 | 0.60 |
|  | 36 | 61 | habitat (urban) | 0.00±0.00 | -0.14 | 0.89 |
|  | 36 | 61 | sex (male) | 0.00±0.00 | 1.21 | 0.23 |
|  | 36 | 61 | age (adult) | 0.00±0.00 | 1.34 | 0.19 |
|  | 36 | 61 | diet*habitat | 0.00±0.00 | 1.04 | 0.30 |

Table S3. Genus-level differential abundance. P values are adjusted for False Discovery Rate (FDR). Values in dietary columns refer to mean relative abundance for each treatment.

|  |  | **pre-dietary manipulation** | | **post-dietary manipulation** | |
| --- | --- | --- | --- | --- | --- |
| **Genus** | **p (FDR)** | **insect** | **seed** | **insect** | **seed** |
| Devosia | <0.001 | 4.24 | 6.48 | 1.93 | 3.06 |
| Rickettsiella | <0.001 | 4.18 | 7.03 | 1.94 | 6.95 |
| Cronobacter | <0.001 | 7.72 | 5.38 | 9.41 | 2.58 |
| Rhizobium | <0.001 | 4.09 | 7.37 | 3.43 | 4.67 |
| Sphingomonas | <0.001 | 6.18 | 7.33 | 3.84 | 6.06 |
| Pantoea | <0.01 | 7.9 | 6.7 | 3.32 | 7.2 |
| Arthrobacter | <0.01 | 7.61 | 6.18 | 4.61 | 5.48 |
| Bradyrhizobium | <0.01 | 6.88 | 7.27 | 4.23 | 4.67 |
| Microbacterium | <0.01 | 3.68 | 4.25 | 3.12 | 6.31 |
| Lactobacillus | <0.01 | 9.58 | 9.16 | 11.51 | 10.12 |
| Bacillus | <0.01 | 4.37 | 3.3 | 2.14 | 5.41 |
| Staphylococcus | <0.01 | 10.21 | 9.86 | 7.07 | 9.72 |
| Brevibacterium | <0.01 | 8.22 | 7.15 | 5.17 | 7.41 |
| Candidatus | <0.01 | 3.09 | 2.92 | 6.24 | 4.84 |
| Serratia | <0.01 | 9.48 | 7.74 | 9.28 | 6.2 |
| Methylobacterium | <0.01 | 10.17 | 11.75 | 14.04 | 12.14 |
| Ureaplasma | 0.016 | 9.3 | 7.73 | 10.26 | 10.71 |
| Brachybacterium | 0.028 | 7.21 | 5.37 | 4.98 | 5.85 |
| Clostridium | 0.032 | 6.02 | 4.44 | 4.36 | 4.02 |
| Rahnella | 0.033 | 9.22 | 8.31 | 7.84 | 6.93 |
| Delftia | 0.035 | 3.09 | 3.28 | 4.78 | 4.77 |
| Carnobacterium | 0.04 | 5.08 | 3.73 | 3.1 | 3.58 |

Table S4. Natural variation in the gut microbiome on Day 1. PSP = Problem Solving Performance.

| **Model** | **total observations** | **Fixed effects** | **β±SE** | **z/t** | **p** |
| --- | --- | --- | --- | --- | --- |
| **Chao1** | 35 | intercept | 18.0±0.93 | 19.40 | <0.01 |
|  |  | PSP | 1.32±1.59 | 0.83 | 0.41 |
|  |  | habitat (urban) | 0.53±1.6 | 0.34 | 0.74 |
|  |  | sex (male) | -1.20±1.55 | -0.78 | 0.44 |
|  |  | age (adult) | 1.65±1.54 | 1.07 | 0.29 |
| **Shannon's index** | 35 | intercept | 2.72±0.22 | 12.1 | <0.01 |
|  |  | PSP | 0.59±0.38 | 1.54 | 0.13 |
|  |  | habitat (urban) | 0.17±0.39 | 0.45 | 0.66 |
|  |  | sex (male) | 0.01±0.38 | 0.02 | 0.98 |
|  |  | age (adult) | 0.20±0.37 | 1.09 | 0.28 |
| **observed species** | 35 | intercept | 0.19±0.21 | 44.50 | <0.01 |
|  |  | PSP | 0.19±0.21 | 0.91 | 0.37 |
|  |  | habitat (urban) | 0.17±0.20 | 0.85 | 0.40 |
|  |  | sex (male) | -0.10±0.20 | -0.50 | 0.62 |
|  |  | age (adult) | 0.18±0.20 | 0.87 | 0.39 |
| **Proteobacteria** | 35 | intercept | -0.37±0.56 | -0.66 | 0.51 |
|  |  | PSP | -0.40±0.76 | -0.53 | 0.60 |
|  |  | age (mature) | 1.27±0.73 | 1.75 | 0.08 |
|  |  | sex (male) | 0.14±0.78 | 0.18 | 0.86 |
|  |  | habitat (urban) | -0.54±0.75 | -0.72 | 0.47 |
| **Bacteroidetes** | 35 | intercept | -6.97±0.44 | -15.77 | <0.01 |
|  |  | PSP | 0.84±0.58 | 1.44 | 0.16 |
|  |  | habitat (urban) | 1.01±0.55 | 1.82 | 0.08 |
|  |  | sex (male) | 0.44±0.57 | 0.76 | 0.45 |
|  |  | age (adult) | 0.65±0.56 | 1.15 | 0.26 |
| **Firmicutes** | 35 | intercept | -3.22±0.27 | -11.97 | <0.01 |
|  |  | PSP | 0.02±0.46 | 0.05 | 0.96 |
|  |  | habitat (urban) | 0.26±0.45 | 0.57 | 0.57 |
|  |  | sex (male) | 0.01±0.45 | 0.02 | 0.99 |
|  |  | age (adult) | -0.48±0.44 | -1.07 | 0.29 |
| **Tenericutes** | 35 | intercept | -5.40±0.51 | -10.60 | <0.01 |
|  |  | PSP | 0.26±0.87 | 0.30 | 0.77 |
|  |  | habitat (urban) | -0.34±0.85 | -0.40 | 0.69 |
|  |  | sex (male) | -0.87±0.84 | -1.04 | 0.31 |
|  |  | age (adult) | 0.28±0.85 | 0.33 | 0.74 |
| **Actinobacteria** | 35 | intercept | 0.00±0.00 | 0.59 | 0.56 |
|  |  | PSP | 0.00±0.00 | -0.06 | 0.96 |
|  |  | habitat (urban) | 0.00±0.00 | **2.47** | **0.02** |
|  |  | sex (male) | 0.00±0.00 | 0.47 | 0.64 |
|  |  | age (adult) | 0.00±0.00 | 1.95 | 0.06 |

Table S5. Beta diversity metrics for (a) natural variation (Day 1); and (b) dietary manipulation. Each beta diversity metric represents a separate model. PSP = Problem Solving Performance.

| **Model** | **Factor** |  | **Jaccard** | **Weighted unifrac** | **Unweighted unifrac** |
| --- | --- | --- | --- | --- | --- |
| a) Natural variation (Day1) | PSP |  | R2=0.03, p=0.40 | R2=0.02, p=0.57 | R2=0.05, p=0.07 |
|  | habitat |  | R2=0.04, p=0.09 | R2=0.06, p=0.09 | R2= 0.04, p=0.09 |
|  | sex |  | R2=0.03, p =0.40 | R2=0.02, p=0.73 | R=0.02, p=0.74 |
|  | age |  | R2=0.03, p=0.30 | R2=0.04, p=0.14 | R=0.04, p=0.13 |
| b) Dietary manipulation (Day 1 &12) | diet |  | R2=0.06, **p<0.01** | R2=0.12, **p<0.01** | R2=0.07, **p<0.01** |
|  | PSP |  | R2=0.02, p=1.1 | R=0.01, p=0.77 | R2=0.04, **p=0.02** |
|  | habitat |  | R2=0.03, **p<0.01** | R2=0.05, **p=0.01** | R2=0.02, p=0.18 |
|  | sex |  | R2=0.02, p=0.35 | R2=0.02, p=0.20 | R=0.01, p=0.92 |
|  | age |  | R2=0.02, p=0.17 | R2=0.03, **p=0.04** | R2=0.02, p=0.38 |
